# Structural and Functional Changes in Biological Systems of Wastewater Treatment Plants Induced by Bicyclic Non-Steroidal Anti-Inflammatory Drugs—A Review

**DOI:** 10.3390/molecules31111828

**Published:** 2026-05-26

**Authors:** Weronika Magdalena Jabłońska, Urszula Guzik, Danuta Wojcieszyńska

**Affiliations:** Institute of Biology, Biotechnology and Environmental Protection, Faculty of Natural Science, University of Silesia in Katowice, Jagiellońska 28, 40-032 Katowice, Poland; weronika.jablonska@us.edu.pl (W.M.J.); danuta.wojcieszynska@us.edu.pl (D.W.)

**Keywords:** bicyclic NSAIDs, wastewater, activated sludge, microbial community, toxicity, changes

## Abstract

The increasing presence of pharmaceutical compounds in aquatic environments poses a significant challenge for wastewater treatment systems worldwide. Among these emerging contaminants, bicyclic non-steroidal anti-inflammatory drugs (NSAIDs) are particularly concerning due to their high consumption, partial metabolism, and long-lasting persistence in wastewater. This review was prepared critically based on popular databases such as PubMed and the Google Scholar website, and using the modern Nested Knowledge platform. The bibliometric analysis was performed using the VosViewer program with the keywords co-occurrence method. The review aims to systematically compile and synthesize current knowledge on the impact of bicyclic non-steroidal anti-inflammatory drugs (NSAIDs) on biological wastewater treatment systems, with particular emphasis on activated sludge. It discusses how these compounds influence microbial community composition, metabolic activity, sludge structure, and overall treatment performance. Furthermore, the distribution of these contaminants in the environment and their degradation efficiency were analyzed. By integrating evidence from both laboratory and industrial studies, this article provides a comprehensive perspective on the environmental risks posed by bicyclic NSAIDs. Our findings also underscore the urgent need for systematic monitoring and adaptive management to mitigate the ecological impact of these widely used pharmaceuticals in the future.

## 1. Introduction

The current challenge for wastewater treatment plants worldwide is developing effective degradation processes to remove micropollutants and other substances that threaten aquatic ecosystems. Micro- and nanoplastics, chemical industrial products, personal care product ingredients, insecticides, and pharmaceutical chemicals are examples of these substances, which are especially significant in mass consumption. Their occurrence in groundwater, surface water, and soils is becoming increasingly common and raises real concerns for the scientific community, wastewater management managers, environmentalists, and governments [1,2,3]. Key research and scientific centers undertake several biotechnological activities aimed at the effective removal of pharmaceutical contaminants, ensuring the best possible water quality, including that of drinking water [4,5]. The presence of pharmaceutical chemicals, designated as emerging contaminants (ECs), leads to negative alterations in the proper functioning of biological processes in organisms exposed to these pollutants, especially those that inhabit aquatic ecosystems [6]. The increased concentration of pharmaceuticals of various types in the environment is indicated as one of the main origins of drug resistance, which is considered one of the most serious contemporary threats to public health [7,8]. Drugs found in aquatic ecosystems include antibiotics, antiepileptic drugs, beta-blockers, and cholesterol-lowering substances.

Nevertheless, a special group of drugs that are an international environmental problem are non-steroidal anti-inflammatory drugs (NSAIDs) [9,10]. NSAIDs are among the most frequently detected pharmaceutical compounds in the aquatic environment worldwide. For nearly 40 years, there has been an interest in them for environmental protection [11]. Literature data indicate that compounds belonging to the NSAID group account for as much as 70% of all micropollutants detected in water [3]. Living beings taking these medications cannot properly metabolize them, resulting in their high concentration in urine and, consequently, in wastewater, indicating their severe environmental impact [6,9].

Current degradation procedures used in most conventional wastewater treatment plants are not entirely effective at removing all NSAIDs from the environment [6]. This is related to the strictly defined physicochemical properties of these drugs, mainly bicyclic NSAIDs with two aromatic rings in their structure. This results in their exceptional stability as chemical molecules, their ability to bioaccumulate, their low water solubility, and their limited sorption [1,10]. Attention should be paid to diclofenac, whose degradation rate in wastewater treatment plants worldwide is only 34% [12]. Conventional wastewater treatment plants use biological processes to deal with environmental micropollutants. Wastewater treatment plants’ biological systems are complex systems that use microorganisms to break down and remove contaminants from urban and industrial wastewater, primarily through biological processes [13]. Modern biological systems use the activated sludge method, which consists of specialized consortia of microorganisms characterized by their metabolic activity. It is an environment that changes dynamically under the influence of many factors, such as the influx of various contaminants and thus changes in the number of substrates for enzymatic reactions. Constant environmental changes lead to differences in the growth rates of microorganisms involved in the breakdown of pollutants [14]. NSAIDs and their toxic secondary metabolites may affect the structure and diversity of the activated sludge microbiota. Aromatic compounds can activate or inhibit enzymes responsible for degrading harmful compounds, thereby altering the metabolic functions of microorganisms and the structure and properties of the biofilm, thereby affecting the efficiency of impurity removal [15]. In addition, under the influence of NSAIDs, the basic operating parameters of the treatment plant are changed, which directly affects the effectiveness of degradation of the drugs in question.

The objective of this review is to provide a coherent and structured synthesis of current knowledge on the effects of environmentally relevant concentrations of bicyclic NSAIDs on biological wastewater treatment systems, with a focus on activated sludge. In particular, the review focuses on how these compounds influence the structure, diversity, and functional stability of microbial communities involved in wastewater treatment processes. Special attention is given to shifts in microbial community composition, changes in metabolic activity, and potential disruptions in key biodegradation pathways.

Additionally, the article evaluates the effects of bicyclic NSAIDs on the efficiency of pollutant removal and transformation processes occurring under different operational conditions in wastewater treatment systems. Furthermore, to outline the scale of the problem, the sources and occurrence of bicyclic NSAIDs in the environment and wastewater treatment plants were presented. By integrating available experimental and environmental studies, the review aims to provide a comprehensive overview of the ecological relevance of these pharmaceuticals and to identify current knowledge gaps regarding their impact on activated sludge systems and related biotechnologies.

## 2. Characteristics of Bicyclic NSAIDs

### 2.1. Mechanism of Action and Classification of Bicyclic NSAIDs in Terms of Their Degradation Potential

**Figure 1:**
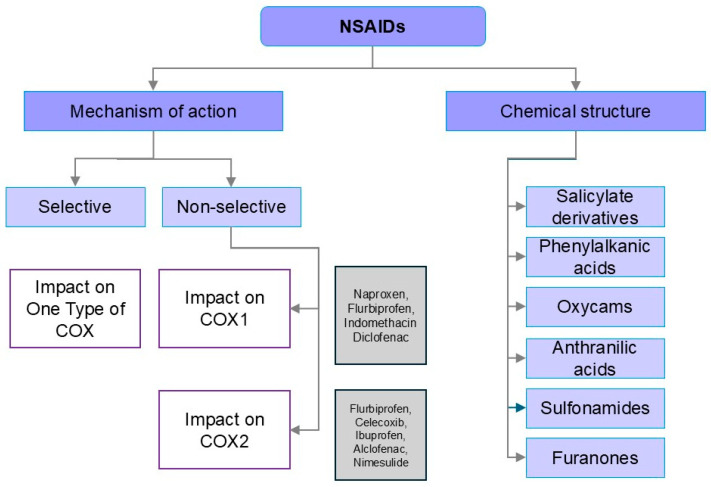
Classification of NSAIDs according to the mechanism of action against COX enzymes and their chemical structure [19,22,23].

Side effects are directly related to the mechanism of action of drugs and what enzymes they interact with [16]. NSAIDs are inhibitors of prostaglandin synthesis (PGs), signaling compounds responsible for inflammation, pain, and fever in the body [17]. In addition to these three functions, prostaglandins cause blood vessels to dilate and swelling to form. Prostaglandin synthesis is inhibited by the inhibition of COX enzymes, including COX-1 and COX-2. COX enzymes are involved in the conversion of arachidonic acid into prostaglandins, as well as into thromboxanes and prostacyclins [18]. An analgesic and anti-inflammatory effect results from blocking the enzyme, thereby preventing the conversion of arachidonic acid to prostaglandins [17,18]. NSAIDs can be classified according to their mechanism of action, which consists of inhibiting two isoforms of cyclooxygenase [19]. Drugs that act on COX-1 affect this enzyme, which is responsible for the physiological production of prostanoids with cytoprotective effects, protecting the gastric mucosa and regulating blood flow. In turn, NSAIDs that act on COX-2 inhibit the inducible isoform of the COX enzyme. The induction of the enzyme occurs in an inflamed state, leading to the release of mediators and the maintenance of the inflammatory response [20,21]. As it was presented by Enthoven et al. [21], NSAIDs are divided into selective, i.e., acting on one type of COX, and non-selective, acting on both COX-1 and COX-2. Most known NSAIDs are COX-1 selective inhibitors, with the added effect of inhibiting platelet aggregation [18]. Bicyclic NSAIDs that inhibit COX-1 include: flurbiprofen, celecoxib, alclofenac, nimesulide, ketoprofen, and ketorolac. On the other hand, bicyclic NSAIDs that inhibit COX-2 include naproxen and flurbiprofen. Diclofenac inhibits COX-2 potently and COX-1 moderately. Aspirin, on the other hand, mainly works by inhibiting COX-1 [22]. The second criterion for classifying NSAIDs is the drug’s chemical structure. There are six main classes of NSAIDs, including those listed by Kyropoulou et al. [23] as (1) salicylate derivatives, (2) phenylalkanic acids, (3) oxycams, (4) anthranilic acids, (5) sulfonamides, and (6) furanones. Figure 1 shows how NSAIDs are categorized into the two groups mentioned above.

Drugs differ in functional groups, which determine their action and therapeutic efficacy. In the context of biological activity, a pharmacophore, like the characteristic arrangement of these functional groups in the molecule, is crucial. Aromatic rings can occur alone or be combined with other rings, forming the core of a molecule and determining its biological and physicochemical properties. The pharmacophore of NSAIDs consists of an aromatic ring and a carboxyl group (-COOH/COO-). NSAIDs differ in their structures and numbers of aromatic rings, which affects their stability and degradation [24,25,26]. Table 1 presents selected bicyclic NSAIDs, along with information on their chemical class and empirical and structural formulas.

Diclofenac (2-(2,6-dichloroaniline) phenylacetic acid) is one of the most widely purchased over-the-counter drugs worldwide, with high durability, which explains its frequent occurrence in sewage. It is classified as a derivative of acetic acid. It dissolves partly in water and partly in a hydrophobic environment. Its structure contains a phenyl ring with two chlorine atoms [37]. Intensive research is underway to develop effective methods for its removal and to assess its impact on the bifunctionality of wastewater treatment plants. Diclofenac is not fully metabolized in the body, and its excretion by the kidneys accounts for up to 65% [26]. The main products of its biotransformation are 4′-hydroxydiclofenac and 5′-hydroxydiclofenac, which are formed primarily through oxidation mediated by CYP2C9 and CYP3A4 and then removed by the kidneys [37]. The remaining 35% of diclofenac is excreted in bile, primarily as acyl glucuronide, which can be epimerized to 2-, 3-, and 4-O-glucuronides.

Naproxen-(2-6-methoxy-2-naphthyl) propionic acid, as an alpha-aryl derivative of propionic acid, also poses a threat to the aquatic environment due to its specific structure containing a naphthalene ring with a methoxy group. The drug’s structure makes it difficult to degrade, and because it is frequently detected in surface and groundwater, the environment is continuously monitored for its presence [38]. It belongs to the acid polar drugs, and its incomplete absorption and metabolic processes in the body cause it to be excreted in the urine, either unchanged or as conjugated metabolites, into the aquatic environment [39].

Flurbiprofen (2-(2-fluoro-4-biphenyl) propionic acid) is one of the three most popular painkillers in some countries, like Turkey [40]. In other countries, its consumption is less common due to the drug’s availability only on prescription [41]. Its concentration in municipal wastewater in Poland ranged from 12 to 51 ng/L [40]. It is extremely insoluble in water, which is why its sodium salt is more commonly used in treatment. It is used for pain associated with cancer and before invasive procedures [42].

Mefenamic acid (N-(2,3-xylyl) anthranilic acid) is a two-ring derivative of diphenylamine, showing poor solubility in water and a high ability to bind with other organic compounds. This characteristic increases its potential for bioaccumulation in the environment. Currently, biological treatment methods are not used for this compound because they are ineffective at degrading it. It is degraded by direct photolysis and photo processes [43,44].

Nimesulide (4-nitro-2-phenoxymethane-sulfonanilide) belongs to the class of sulfonanilides [45]. It contains a nitro group at the fourth position of the aniline ring, and a phenoxymethane sulfonanilide structure. It dissolves poorly in water. Its mechanism combines anti-inflammatory effects with free radical inhibition. In addition, it inhibits histamine release from mast cells and basophils and regulates apoptotic processes in chondrocytes and connective tissue cells [46].

Many NSAIDs occur commercially in two possible forms: (1) sodium salt or potassium salt and (2) free acid. The differences between the two forms affect their water solubility and bioavailability. NSAIDs are also found as prodrugs [47]. Both salt, acid, and prodrug forms of NSAIDs are detectable in surface water [48]. Acid drugs are mainly found in ionic form [49]. The physicochemical features of a pharmaceutical, including solvation, dissociation, and phase division, are principally in charge of its fate in both the body and the aquatic environment [50,51].

### 2.2. Sources and Environmental Occurrence of Bicyclic NSAIDs

Regulations on the sale of bicyclic NSAIDs vary by region, determining their availability and, by extension, their consumption. The sale of drugs and their mass release into the environment is determined by the availability of the drug without a prescription [52]. Prediction of NSAIDs occurrence in the environment is limited by the inability to record over-the-counter drug purchases. Region, culture, traditions, and lifestyles influence the preferences and consumption of NSAIDs. For example, bicyclic NSAIDs are significantly less used by residents of China than by residents of Europe, the USA, and Canada [53,54]. The highest per capita concentrations of diclofenac were estimated in Spain and Finland, where its use varied with the season of the year [54].

Due to the dynamic development of the pharmaceutical industry, the widespread availability of drugs and their increasingly frequent use and abuse in human and veterinary medicine, as well as the penetration of these substances and their metabolites into the environment, the concentration of bicyclic NSAIDs in the environment continues to increase year by year [6,9]. Excreted drugs enter municipal, hospital, and industrial wastewater. Medicines also enter the environment through waste [55,56]. Inadequate segregation of drugs as waste, incomplete disposal, and ineffective methods of removing drugs from the environment are also crucial for the existing concentrations of drugs in water [56,57]. A significant source is municipal wastewater, since consumed drugs are excreted in urine and end up in sewage systems, and the elimination of unused drugs in household sewage leads to the rise in pharmaceutical loads [9,11]. Another substantial component is hospital effluent, which is sometimes released directly into sewage systems due to the high volume of medications consumed [55]. Since chemical components can enter soil, surface waters, and wastewater due to improper disposal, inadequate sanitation, or incorrect segregation, pharmaceutical waste and waste generated by the pharmaceutical industry also make major impacts. Furthermore, when veterinary medications are overused in livestock husbandry, their active substances are excreted in animal urine and feces, causing them to get into sediments and surface streams and to gather up in aquatic ecosystems and agricultural-generated products [57,58].

Ecotoxicology is an interdisciplinary field of science that currently focuses on, among other things, monitoring the concentrations of pharmaceuticals in the world’s waters, estimating the concentration of pharmaceuticals entering wastewater treatment plants, and studying the impact of xenobiotics on ecosystems [59,60]. It was estimated that more than 1000 tons of diclofenac is being consumed annually [61]. Therefore, bicyclic NSAIDs are an important issue for ecotoxicology. The concentrations of NSAIDs in the waters vary. However, bicyclic NSAIDs in aqueous environments often have low concentrations, expressed in units such as ng/L to μg/L [62].

Many scientific reports indicate different concentrations of bicylic NSAIDs in water. Studies conducted in Brazil have shown that even 0.2 μg/L of diclofenac and ibuprofen in water disrupts the development and hormonal profile and affects the behavior of fish [63]. According to the paper by Skocovska et al. [64], naproxen and diclofenac were found in several river samples. The Stěnava River had the highest naproxen concentration at 160 ng/L, followed by the Borecký Stream in Souvrať at 110 ng/L, and the Cidlina and Výmola rivers at 69 and 58 ng/L, respectively. The concentration of diclofenac in the Výmola and Cidlina rivers was approximately 260 ng/L, while the concentrations in the Chrudimka and Stěnava rivers were 200 ng/L each. The studies by Mainero Rocca et al. [65] showed that nimesulide was detected at 9 ng/L in the Cremera River and naproxen at levels less than 3 ng/L in the Vetoio River, whereas nimesulide was detected at the same concentration in this river. The amount of ketoprofen in the Bracciano River was 30 ng/L. Significant regional diversity in bicyclic NSAIDs concentrations is evident in the data reported by Antos et al. [66] for surface waters across Europe. Diclofenac, for instance, was found in several countries. The Saronikos Gulf and Elefsis Bay in Greece’s coastal waters had comparatively low amounts (ranging from 1.4 to 16.3 ng/L), whereas the Pärnu River in Estonia had intermediate levels (ranging from 11 to 53 ng/L). The Rokitnica River in Poland had diclofenac concentrations ranging from 12 to even 2200 ng/L, while the Tollense River in Germany had quantities as high as 350 ng/L. Similarly, the concentration range of ketoprofen in the Tollense River was broad, ranging from 0 to 2.13 ng/L. Ketoprofen concentrations in the Rokitnica River in Poland ranged from 2.4 to 280 ng/L, while the Leba River in the Czech Republic was reported to have a concentration of 929.8 ng/L. In addition, detectable levels of ketoprofen were discovered in the Ebro River in Spain (stage 70 ng/L) and the Pärnu River in Estonia (stage > 2.1 ng/L). Naproxen levels in the Rokitnica River in Poland and the Danube River in Hungary ranged from 5.7 to 62 ng/L. Higher levels of this medication were found in surface waters in Slovenia (range: 17–80 ng/L), the Warta River in Poland (100 ng/L), and the Aisonas River in Greece (72 ng/L). In several freshwater bodies, fenoprofen was detected at concentrations ranging from 2 to 54 ng/L in German rivers and up to 84 ng/L in Polish rivers [66].

### 2.3. Concentrations of Bicyclic NSAIDs in Wastewater Treatment Plants

Pollutants in wastewater treatment plant inflows come from municipal, industrial, and hospital wastewater and carry high concentrations of contaminants, including bicyclic NSAIDs. After treatment, wastewater discharges into rivers, lakes, and groundwater, making the final drug concentration in the wastewater treatment plant effluent extremely important [63,67]. According to Näslund et al. [68], the average naproxen levels at the wastewater treatment plant ranged from 121 ng/L in the effluent to 1674 ng/L in the influent. In Canada, naproxen residue concentrations in the effluent reached a significant level of 33.9 μg/L before 2006. Similar naproxen concentrations were also reported in the effluent of wastewater treatment plants in Sweden [69]. For diclofenac, Näslund et al. [68] report equivalent values of 149 ng/L in the effluent and 196 ng/L in the influent. The research by Araujo et al. [49] shows that the content of diclofenac in the influent was 990 ng/L, while in the effluent, these levels turned out to be lower: 560 ng/L and 365 ng/L for diclofenac. Another study byYan et al. [54] showed that in Guangzhou wastewater treatment plants, 31.5 ng/L to 54.6 ng/L of diclofenac was detected in one wastewater treatment plant, and 45.3 ng/L to 86.3 ng/L in the second one in the same town. In Brazil, in the Sao Paulo region, the concentration of all NSAIDs in wastewater treatment plants ranged from 2.5 ng/L to 2094.4 ng/L [63]. Araujo et al. [49] declared that the concentration of ketoprofen in the influent was 0.18 μg/L, and in the effluent, the concentrations of this drug were estimated from 0.554 μg/L to 0.743 μg/L. A study by Aydin et al. [70] reported a broad spectrum of analgesics and anti-inflammatories in hospital wastewater in Turkey, depending on the season. The highest average concentrations in hospital wastewater in the summer were recorded for ketoprofen (9193 ng/L), while the concentration of naproxen at that time was 7186 ng/L. Gallardo-Altamirano et al. [71] expressed the NSAIDs wastewater load as the daily mass load of pharmacological compounds per 1000 people. In this way, the daily amounts of bicyclic NSAIDs in the incoming and outflowing wastewater during the two phases of operation of the biological wastewater treatment plant system in Spain were determined. In the first phase, the concentration of diclofenac in the influent was 291 mg/(1000 inh×d), while in the effluent it reached 257 mg/(1000 inh×d). In the second phase, these values were 246 and 285 mg/(1000 inh×d), respectively, indicating ineffective removal of this compound. The concentration of naproxen in the first phase, in the influent was 1748 mg/(1000 inh×d) and in the effluent 901 mg/(1000 inh×d), while in the second phase of purification, the level of naproxen decreased from 1636 to 211 mg/(1000 inh×d). In the case of ketoprofen in phase one, the amount of drug in the influent and effluent did not differ significantly (234 to 259 mg/(1000 inh×d). In the second phase, the amount of ketoprofen was 294 mg and decreased to 166 mg (1000 inh×d) [71].

The comparison presented in Table 2 indicates significant heterogeneity among studies regarding analytical methodologies, sampling strategies, and wastewater treatment setups. Most studies used LC-MS/MS or related chromatographic techniques due to their high sensitivity in detecting trace amounts of drugs. However, differences in sampling periods (seasonal versus routine monitoring), matrices (surface water, influent, wastewater, hospital wastewater), and treatment technologies (conventional activated sludge, hospital-specific treatment) make direct comparison of reported bicyclic NSAID concentrations difficult. Seasonal consumption patterns, local prescribing practices, and treatment efficiency further contribute to the observed regional variability.

## 3. Effects of Bicyclic NSAIDs on Aquatic Organisms

Bicyclic NSAIDs are difficult to degrade and are often detectable in groundwater, surface water, as well as in the inflows and outflows of wastewater treatment plants, posing a real threat to non-target organisms, including fish, crustaceans, arthropods, protists, bacteria, and fungi inhabiting these ecosystems [10,75,76]. Among these organisms are also microorganisms that form activated sludge consortia in the biological systems of sewage treatment plants [77,78]. Näslund et al. [68] report that bicyclic NSAID concentrations above 4.6 μg/L in water are considered hazardous to aquatic organisms. The elaboration by Wojcieszyńska and Guzik [10] indicates that naproxen is highly toxic to organisms such as *Vibrio fisheri*, both in its unchanged form and in photodegraded form. The authors emphasize the higher toxicity of the drug in its metabolized form than in the original form, due to changes in the structure and molecular weight of degradation products occurring in the transformation process. Osorio et al. [79] also evaluated the toxicity of bicyclic NSAIDs, diclofenac unchanged and its nitrogenous forms, to *Daphnia magna* and *V. fischeri*. The EC_50_ value for diclofenac was 53.9 mg/L for *D. magna* and 22.9 mg/L for V. fischeri. Its nitro-diclofenac compound was more toxic to *V. fischeri* (EC_50_ = 11.7 mg/L), while *D. magna* toxicity was lower (EC_50_ = 86.7 mg/L). Naproxen and its metabolites disrupt the endocrine system, metabolism, and mRNA expression in fish. Studies show that naproxen and its metabolites have a toxic effect on crustacean growth. Toxic concentrations to these organisms are about 10 ug/L. Schwaiger et al. [80] observed marked kidney damage and degenerative and necrotic changes in gill support cells in *Oncorhynchus mykiss* they studied, which were exposed for 28 days to diclofenac at concentrations of 1, 5, 20, 100, and 500 μg/L. Hernández-Zamora et al. [81] showed complete growth inhibition of *Pseudokirchneriella subcapitata*, reproductive and metabolic disorders in *Daphnia curvirostris*, as well as an increased mortality and sublethal effects in *Danio rerio* following exposure to diclofenac. Against carp, naproxen at 10 μg/L delayed hatchability and inhibited hatchability [82]. Analyses of the effect of flurbiprofen on *D. magna* and *Aliivibrio fischeri* have shown its acute toxicity. In *D. magna*, the EC_50_ was estimated to be 10.74 mg/L after 24 h and 5.89 mg/L after 48 h. For *A. fischeri*, EC_50_ after 15 min was 1.9 mg/L [83]. In addition, toxicological studies are regularly conducted on activated sludge bacteria in wastewater treatment plants; for example, these studies show that diclofenac adversely affects microorganisms by damaging their cellular structure. This effect becomes noticeable at concentrations from 5 μg/L [84].

## 4. Methodology of Systematic and Critical Literature Review

**Figure 2:**
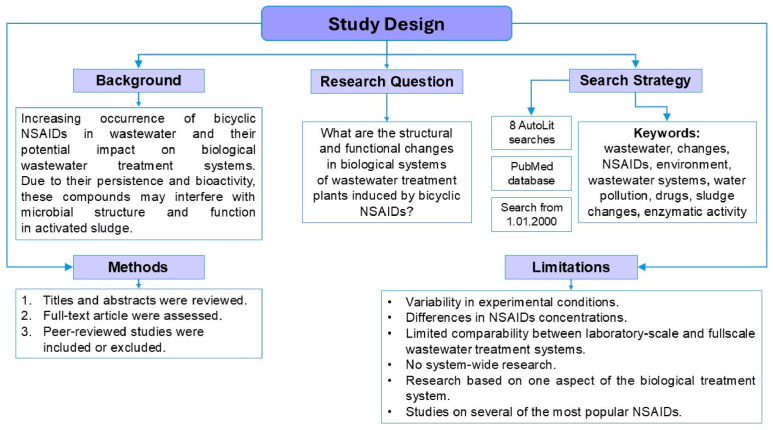
Systematic review protocol.

To evaluate the main topic of this article, both a critical and systematic literature review were conducted in accordance with the PRISMA guidelines in the PubMed database, using the modern Nested Knowledge (https://nested-knowledge.com) platform. A systematic literature review protocol has been prepared on the platform, focusing on the purpose of the meta-analysis, determining the scope of searched research, defining keywords, the method of excluding research, and the allowed types of searched research papers. The search protocol is shown in Figure 2.

Searches were conducted on the AutoLit platform using the “Create New Nest” option and the appropriate keywords for each search. A total of 8 searches were applied to one Nest. The search on the platform was automatically performed by the system using the PubMed database. The analysis of abstracts and full text versions included applying the inclusion and exclusion criteria formulated in the protocol and implemented in the system. Inclusion criteria included experimental studies on the effects of bicyclic NSAIDs on biological system components in wastewater treatment plants and publications after the year 2000. To search for research papers in the AutoLit system, the following keywords were used: wastewater, changes, NSAIDs, environment, wastewater systems, water pollution, drugs, sludge changes, and enzymatic activity. The exclusion criteria included: the studies that did not concern bicyclic NSAIDs, the studies that did not closely correlate with the subject matter of the work, the lack of connection with the subject matter of biological wastewater treatment systems, the nature of the review work, the studies that did not present changes in biological treatment systems, and the studies that did not concern drugs from the NSAID group.

**Figure 3:**
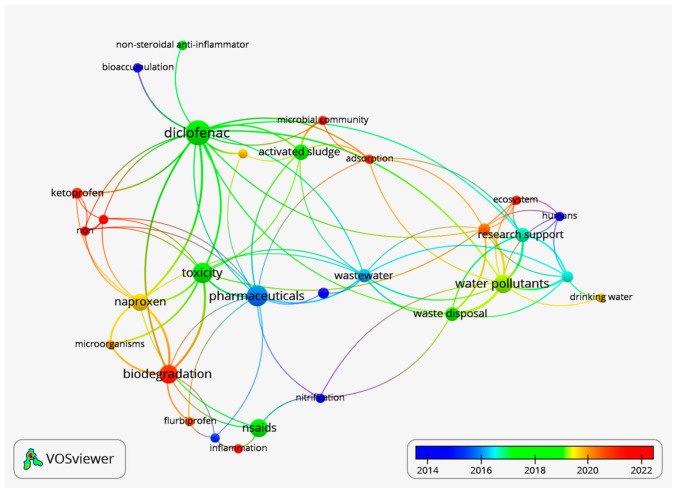
Bibliometric analysis of keywords and their co-occurrence carried out using VOSviewer software.

To conduct a critical review of the literature, the authors independently searched the PubMed, Scopus, and Google Scholar databases for research papers. An additional bibliometric study in this paper, visualized in Figure 3, was carried out using VOSviewer software (version 1.6.20) with the co-occurrence of the keywords method. The analysis was based on all the literature papers used in this review, using bibliographic data in the RIS (Research Information System) format. This analysis was based on full counting. Each occurrence of a given keyword in the publication was counted with equal weight. The normalization method used was the association strength method, which allows the identification of real thematic relationships between keywords by correcting for the frequency of their co-occurrence relative to their individual frequencies. The minimum number of occurrences of a keyword is set at 3. Out of all keywords, 40 met the set threshold, so a maximum of 40 terms with the highest total binding strength were selected for further analysis and visualization.

## 5. Discussion of Results

### 5.1. Results of a Systematic Literature Review

**Figure 4:**
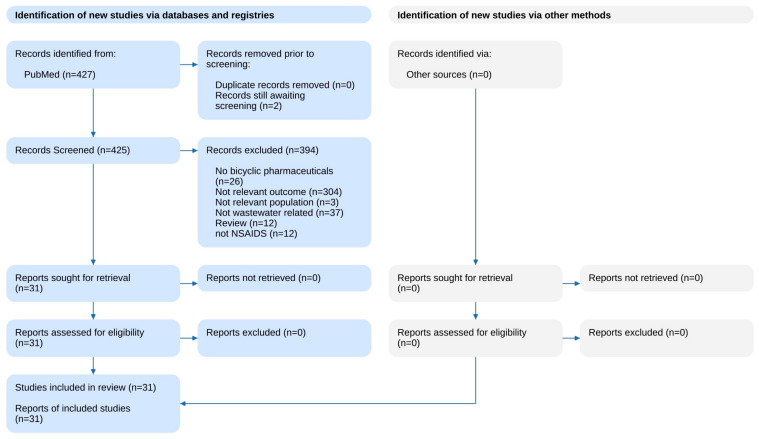
PRISMA diagram [https://nested-knowledge.com/].

As part of a systematic literature review, a PRISMA diagram was created according to the 2020 formula. The PRISMA diagram illustrates the steps in identifying, selecting, and incorporating studies into the analysis (Figure 4).

As a result of searching the databases, 425 records were identified, and no additional publications from other sources were entered into the system. The nested knowledge system automatically rejected duplicates of searched works, so their number does not appear on the PRISMA diagram. All 425 works were then subjected to the initial screening stage. The first analysis of the works focused on the titles and abstracts of the works, and then each article was analyzed in full text. At the stage of title and abstract evaluation, 394 publications were excluded. The main reasons for exclusion included: lack of pharmaceutical compounds with a bicyclic structure (n = 26), lack of correlation with the subject matter of the paper (n = 304), inadequate population of parameters (n = 3), lack of connection with the subject matter of biological wastewater treatment systems (n = 37), nature of the review work (n = 12), and lack of reference to bicyclic NSAIDs (n = 12). A total of 31 articles were included in the full text analysis in this paper. Of the selected 31 subsequent articles, six had to be rejected for the review paper due to a lack of access to their full texts in the databases.

The resulting map (Figure 4) of bibliometric analysis, which is based on keywords and their co-occurrence, reveals a clearly structured network of concepts, in which the terms “pharmaceuticals”, “toxicity”, “wastewater”, “biodegradation”, and “activated sludge” occupy a central position, indicating the dominant directions of research on the presence and impact of pharmaceuticals in biological wastewater treatment systems. Bicyclic NSAIDs, such as diclofenac, naproxen, and ketoprofen, among others, form a strongly correlated separate cluster with the concepts of “activated sludge”, “microbial community”, “wastewater treatment”, “waste disposal”, “bioaccumulation”, and “biodegradation”. This shows that research on these substances focuses not only on their presence in wastewater, but also on their impact on the microbiological structure of activated sludge and the course of key biological processes. The strong associations between the terms “diclofenac” and “naproxen” with the terms “toxicity” and “bioaccumulation” indicate that these compounds are seen as particularly problematic due to their durability, limited susceptibility to biodegradation, and potential for bioaccumulation in organisms. The presence of concepts such as “nitrification” and “environmental risk assessment” in the vicinity of the terms drugs suggests that the impact of bicyclic NSAIDs on ecosystem functions and technological processes, including sensitive steps in biological nitrogen removal, is increasingly being analyzed. The obtained map structure confirms that bicyclic NSAIDs are an important factor in environmental pressure on biological wastewater treatment systems, while simultaneously influencing their structure and functioning. The central location of these compounds in the network of concepts and their strong links to toxicological and technological categories could indicate a potential need for further research into the mechanisms of adaptation and destabilization of activated sludge under the influence of bicyclic NSAIDs. Thanks to the obtained map of the co-occurrence of keywords, it was proven that this literature review is necessary to draw basic conclusions regarding the effect of bicyclic NSAIDs on the structure and functionality of biological wastewater treatment systems.

### 5.2. Bicyclic NSAIDs Degradation in Biological Systems of Wastewater Treatment Plants

Among the wastewater treatment methods currently applied, biological treatment systems are among the most widely used. These systems are based on the metabolic activity of microorganisms and are primarily designed to remove organic matter and a wide range of micropollutants [85]. In biological wastewater treatment, activated sludge, biofilm, or granular sludge technologies are employed. Activated sludge is a complex microbial consortium, mainly composed of bacteria embedded in extracellular polymeric substances (EPS) and associated with inorganic particles, forming flocs that degrade pollutants [14,85]. The process is carried out in bioreactors under controlled conditions that promote microbial growth, while solid–liquid separation leads to the formation of effluent, return sludge, and excess sludge [86,87]. Overall, biological treatment is considered a cost-effective and environmentally safe technology when properly designed [73,88]. Within these systems, a wide range of organic micropollutants, including pharmaceuticals such as bicyclic NSAIDs, undergo transformation processes. These compounds are not completely removed but are instead converted into more polar and often simpler products, which typically increases their aqueous solubility and reduces their bioaccumulation potential and environmental persistence [89,90]. This transformation is primarily driven by microbial activity and occurs through either direct metabolism or cometabolism involving both heterotrophic and nitrifying bacteria. In particular, key enzymatic systems such as cytochrome P450 monooxygenases—responsible for hydroxylation of aromatic structures—and ammonia monooxygenase (AMO), which is especially active under nitrifying conditions and long sludge retention time (SRT), play a central role in these processes [91,92,93]. The efficiency and direction of these reactions depend on operational parameters, sludge age, and microbial community composition [94,95,96].

The actions that occur in activated sludge are biotransformation and sorption. Sorption is governed by the physicochemical properties of the compounds, such as hydrophobicity and acid–base behavior, as well as environmental conditions, including pH and ionic strength [94,95]. In this context, hydrophobic pharmaceuticals may associate with lipid fractions of biomass, including microbial cell membranes, or adsorb onto sludge particles, and this behavior can be described using distribution coefficients (Kd) [94]. Bicyclic NSAIDs may also undergo a range of specific biotransformation reactions. The most common reactions include hydroxylation of aromatic rings, O- and N-demethylation, oxidation and reduction of functional groups, decarboxylation, and dechlorination [86,92,93].

**Figure 5:**
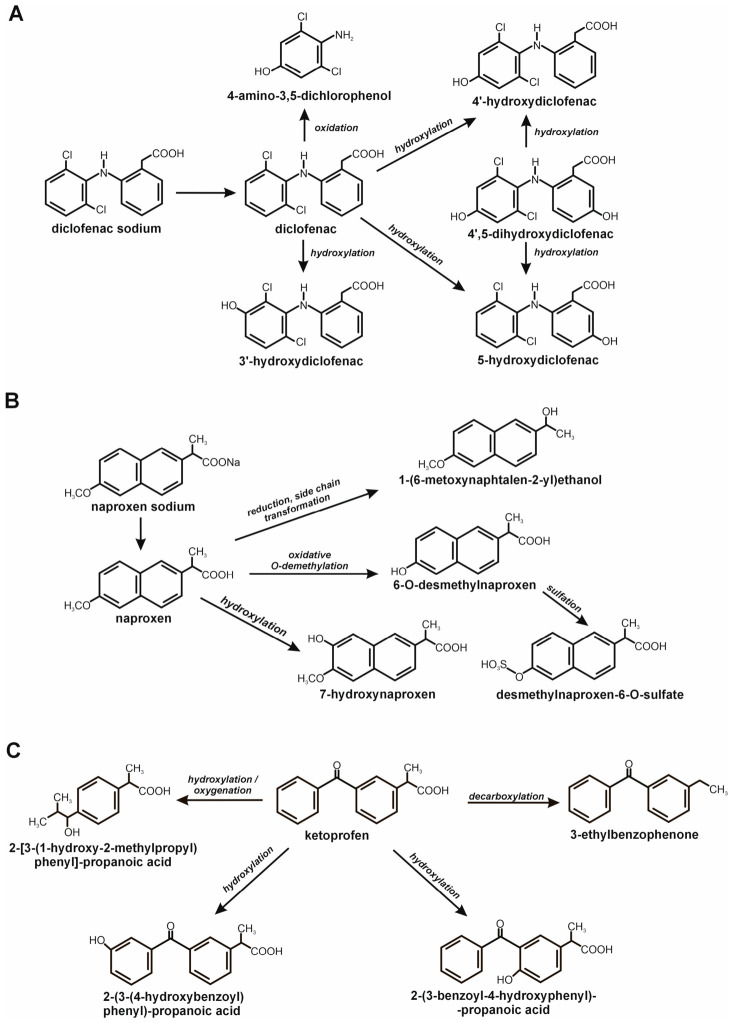
Schematic overview of the dominant biotransformation pathways of diclofenac (**A**), naproxen (**B**), and ketoprofen (**C**) [10,29,91,99,100,101,102,103,104,105,106,107,108].

Focusing on individual compounds, diclofenac is one of the most intensively studied bicyclic NSAIDs in biological treatment systems due to its persistence. Its biotransformation proceeds through reactions such as hydroxylation of aromatic rings, amidation and deamidation of intramolecular molecules, decarboxylation, oxidation, reduction, and chlorine removal (Figure 5A) [86,97,98].

It has been shown that diclofenac hydroxylation leads to the formation of 3′-hydroxydiclofenac, 4′-hydroxydiclofenac, 5-hydroxydiclofenac, and 4′,5-dihydroxydiclofenac, mainly via cytochrome P-450 enzymes. In turn, diclofenac oxidation yields 4-amino-3,5-dichlorophenol [79,86,97,98]. Although hydroxylation is the dominant pathway for the biotransformation of diclofenac, nitrosation and nitration products, as well as further oxidation reactions and structural remodeling of the molecule, have also been identified in the activated sludge [79]. These processes lead to partial cleavage of diclofenac with the formation of dichlorobenzoic acid and to the modification of the side chain, resulting in the formation of benzyl alcohol derivatives. In addition, some of the detected metabolites indicate intramolecular cyclization and subsequent oxidation steps, leading to the formation of 1-(2,6-dichlorophenyl)-1,3-dihydro-2H-indole-2-one [79].

The biotransformation of diclofenac can occur via bacterial and fungal action, as documented in numerous scientific papers [91,99,100]. Domaradzka et al. [91] described that the biotransformation of diclofenac to 4-(2,6-dichlorophenylamino)-1,3-benzenedimethanol occurred with the participation of the fungus *Trametes versicolor*. In addition, the authors indicated that diclofenac may also undergo reactions catalyzed by manganese peroxidase from *Phanerochaete chrysosporium* and *P. sordida* YK-624. Carneiro et al. [93] indicate that the biotransformation of diclofenac results from non-specific reactions catalyzed by enzymes of nitrifying bacteria, especially ammonium monooxygenase, which participate in the cometabolism of various organic compounds. Bouju et al. [101] indicated that 4-hydroxyclofenac is an important marker of hydroxylation processes in biological purification systems, and that the high precipitate retention time in MBR reactors promotes a more efficient transformation of this drug and its metabolites.

Similarly, naproxen undergoes both sorption and biotransformation. Approximately 23% of the compound may be associated with activated sludge biomass, while the remaining fraction is biologically transformed [91]. The main metabolic route also involves hydroxylation, but catalyzed by cytochrome P450 enzymes or laccases, leading to the formation of 7-hydroxynaproxen (Figure 5B). In turn, Carneiro et al. [93] reported that the biotransformation of naproxen occurs by O-demethylation of naproxen to desmethylnaproxen. Microorganisms of the genus *Pseudoxanthomonas*, among others, are responsible for the course of this reaction. Desmethylnaproxen can further undergo phase II conjugation reactions, such as sulfation to form desmethylnaproxen-6-O-sulfate, as observed in fungal systems (e.g., *Cunninghamella* spp.) [108].

Fernandez-Fontaina et al. [107] demonstrated that naproxen undergoes efficient biological transformation in systems characterized by active nitrification. Under these conditions, the dominant metabolic pathway involves the reduction of the carboxyl functional group, leading to the formation of 1-(6-methoxynaphthalen-2-yl)ethanol. The highest transformation rates were observed in reactors operating under complete nitrification, involving both ammonia- and nitrite-oxidizing bacteria. This effect was attributed to the enhanced activity of ammonium monooxygenase, an enzyme with low substrate specificity, particularly under ammonia-limited conditions. Conversely, in environments where heterotrophic microorganisms predominated, naproxen removal was considerably less effective, yielding mainly a single detectable metabolite derived from carboxyl group reduction [107].

Ketoprofen, in turn, follows a slightly different but equally complex transformation pattern. Its degradation occurs through bacterial, fungal, and enzymatic oxidative, reductive, and hydrolytic reactions (Figure 5C). The initial step is hydroxylation of the aromatic ring, producing (3-hydroxyphenyl)(phenyl)methanone [29]. This is followed by additional hydroxylation and carbonyl group reduction, yielding intermediates such as 3-(1-hydroxyphenyl)methanone. Concurrently, oxidation of the propanoic acid side chain produces derivatives including 2-(3-hydroxy-(hydroxymethyl)phenyl)propanoic acid and 2-(3-carboxycarbonylphenyl)-3-hydroxypropanoic acid. Further reactions, including demethylation and alkyl chain shortening, lead to compounds such as (3-ethylphenyl)(phenyl)methanone and (3-hydroxyphenyl)(oxo)acetic acid, and ultimately to aromatic ring cleavage and mineralization to CO_2_ and H_2_O [29,91].

Described compound-specific pathways illustrate that bicyclic NSAID removal in activated sludge systems is governed by interconnected biological and physicochemical processes rather than isolated reactions. In this context, microbial community composition plays a crucial role in determining transformation efficiency. Reported organisms involved in NSAID biotransformation include bacterial genera such as *Pseudomonas*, *Rhodococcus*, *Klebsiella*, *Brevibacterium*, *Raoultella*, *Planococcus*, *Stenotrophomonas*, and *Labrys*, as well as fungi such as *Trametes versicolor* and *Pleurotus* spp. [109]. Overall, the fate of NSAIDs in activated sludge systems is determined by the combined action of sorption processes and enzyme-mediated biotransformation pathways.

### 5.3. Functional and Structural Changes in Biological Wastewater Treatment Systems

The impact of bicyclic NSAIDs on the biological functioning of activated sludge, including the structure of the microbiome, the stability of nitrification and denitrification processes, EPS production, and microorganism adaptability, is gaining attention in the scientific literature [87,91,92,93,110]. This review gives particular attention to comparisons between laboratory and industrial data, to the evaluation of the biological effects of drugs as a function of concentration, and to the identification of adaptation mechanisms in activated sludge bacteria. According to studies conducted in wastewater treatment plants, the presence of bicyclic NSAIDs in the environment usually does not disturb basic technological processes [111]. To illustrate and systematize the considerations, two definitions of the impact of bicylic NSAIDs were used: the impact on functional changes and the impact on structural changes in biological wastewater treatment systems. It was assumed that functional changes include parameters such as nitrification and denitrification processes, phosphorus removal, organic carbon removal, enzymatic activity, hydrolysis and aerobic consumption, drugs metabolism and biodegradation, oxidative stress, and resistance of microorganisms. Structural changes, on the other hand, include biomass mass and quality, changes in bacterial consortia, and changes in sludge structure.

### 5.4. The Effect of Bicyclic NSAIDs on the Metabolic Activity and Functional Stability of Activated Sludge

The accumulation of bicyclic NSAIDs in a wastewater treatment plant’s influent can affect the effectiveness of their elimination in activated sludge systems, thereby impacting the course of metabolic pathways and the activity of microorganisms responsible for these processes. Nevertheless, some research suggests that the activated sludge bacterial community maintains its functionality at environmental NSAIDs dosages, and the alterations that occur are compensatory rather than hazardous. According to research by Zhao et al. [87], Symsaris et al. [112], Jiang et al. [110], and Dolu and Nas [113], bicyclic NSAIDs may inhibit their own degradation and reduce the effectiveness of certain biological processes in wastewater treatment plants.

Both the structure and microbial composition of activated sludge, as well as certain degradation methods, can be changed by the presence of pharmaceuticals in wastewater treatment plants [110,112,114]. This impact depends on concentration and selectivity. According to data from Dolu and Nas [113], bicyclic NSAIDs preferentially interfere with biodegradation pathways, which decreases activated sludge’s ability to operate effectively. The low elimination of diclofenac (7.9–52.2%) and the high removal efficiency of naproxen (nearly 99%) demonstrate that not all compounds go through total mineralization under the same technological setting, suggesting the enzymatic limitations of microorganisms toward more complex chemical structures. Jiang et al. [110] found that a combination of 5 µg/L each of diclofenac, ibuprofen, and naproxen reduced the elimination effectiveness of pharmaceuticals in SBRs. A substantial decrease was noted for diclofenac, with its elimination rate decreasing from 64% in a system containing only this compound to 57% when all three drugs were combined. These findings might suggest that drug mixing may result in competing for biotransformation pathways or reduced enzymatic accessibility in the sludge’s microorganisms. Studies by Symsaris et al. [112] showed that the methanogenic community was reorganized and that methane generation was inhibited in a dose-dependent manner. Complete inhibition of methanogenesis at 3000 mg/L diclofenac and a 31% larger decrease in methane generation following an increase in biomass, demonstrate that the effect is caused by the direct influence of pharmaceuticals on the metabolic process rather than by an inadequate number of microorganisms. Limited removal efficacy could be attributed to both the chemical stability of compounds with substituted aromatic rings and their attraction for activated sludge matrix elements such as EPS and cell lipid fractions. In biological systems, altered biotransformation processes driven by biochemical competition can also result in prolonged persistence of more difficult-to-eliminate structures. [87,98,113,115]. Wu et al. [98] observed a faster hydroxylation of diclofenac under nitrifying conditions, leading to the decomposition of approximately 84% of diclofenac within 48 h, while in heterotrophic sludge, the decomposition was below 50%. The findings suggest that the initial transformation of diclofenac is strongly dependent on microbial community structure. However, the formation of specific transformation products under nitrifying conditions indicates that additional degradation could require the presence of diverse heterotrophic microbes with broader enzymatic activity. This could help clarify the accumulation of intermediates identified in the Dolu and Nas [113] studies, as well as the decreased removal efficiencies reported in mixed pharmaceutical systems by Jiang et al. [110]. This means that the total degradation of bicyclic NSAIDs involves interactions among several functional groups of microorganisms. Demaria et al. [116] confirmed this connection in a laboratory membrane bioreactor (MBR), where, after the introduction of 2 mg/L diclofenac, the initial elimination rate was low, pointing to a period of adaptation and selective restructuring of the community of bacteria. Variable degradation efficiency resulted from subtle changes in bacterial structure, underscoring the role of the drug’s presence in assessing the biomass’s operational profile. Simultaneously, it was demonstrated that an important improvement in removal efficiency resulted from extending the accessibility of an easily absorbed carbon source. It has been shown that exposure of biomass to bicyclic NSAIDs such as diclofenac is associated with inhibition of enzymatic activity, induction of oxidative stress, changes in microbial community structure, and a reduction in intracellular energy reserves [30,87,110,117,118,119,120]. Consequently, it might seem that these pharmaceuticals are decreasing the effectiveness of biological elimination methods. However, the results of Kołecka et al. [120] indicate that bicyclic NSAIDs do not have a toxic or destructive effect on the wastewater treatment process. In a study by Kołecka et al. [120] that was based on an SBR treatment plant, no deterioration in the operating parameters of the installation was observed, which is consistent with the observations of Jiang et al. [110], where, despite reduced elimination of pharmaceuticals, the stability of biological processes was maintained. Kołecka et al. [120] also showed that the efficiency of diclofenac degradation increased in the presence of an additional source of easily assimilable carbon, confirming the importance of cometabolism and a diverse consortium of microorganisms for the complete transformation of bicyclic NSAIDs.

Kołecka et al.’s [120] research supports the outcomes of Dolu and Nas [113] and Wu et al. [98], revealing that pharmaceutical degradation is restricted by inadequate metabolic and structural conditions in microbial communities. Additional evidence for the selective impact of bicyclic NSAIDs on activated sludge is provided by the study by Hashim et al. [111], who did not observe any significant functional disturbances of the entire system at low drug concentrations. The study showed that under MBR conditions, bicyclic NSAIDs are biodegraded enantioselectively. Naproxen and ketoprofen were removed by 38–47% and 67–70%, respectively. In the case of naproxen, a clear change in the enantiomeric fraction and a simultaneous increase in the concentration of the (R) enantiomer in the permeate were observed, indicating chiral inversion occurring in the active sludge. Although there were noticeable modifications in metabolism, there were no significant modifications in TOC, total nitrogen, pH, conductivity, or turbidity. This indicates that the presence of bicyclic NSAIDs at the measured concentrations had a selective impact on the metabolic pathways that metabolized them, rather than impairing the overall biological system’s capacity to function as usual. In conclusion, bicyclic NSAIDs elimination in activated sludge is highly selective and depends on the compound’s dosage, the microbial community’s ability to adapt, and the availability of other substrates [111].

The rate of cellular respiration, most frequently defined as the oxygen uptake rate (OUR), is a direct indicator of treatment plant performance in aerobic conditions. It is believed that a decrease in the rate of aerobic respiration indicates reduced metabolic activity of biomass [121]. By examining the kinetic parameters of microbial growth, such as the maximal growth rate, changes in metabolic activity can also be assessed. A decrease in the maximum growth rate of nitrifying bacteria indicates a decline in their metabolic efficiency [118,122]. Literature data on the effects of bicyclic NSAIDs on nitrification and denitrification processes are inconsistent. Some authors did not observe significant changes in nitrification efficiency or overall biological functions [111,114,117,118,122,123]. However, other studies have shown inhibition of nitrification and denitrification under oxidative stress and selection pressure in the presence of low concentrations of bicyclic NSAIDs [38,79,87,110]. Increasing diclofenac concentration to 2 mg/L resulted in significant nitrification suppression, as evidenced by a slowing of ammonia oxidation and an increase in effluent NH_4_^+^ concentration. Peak quantities of NO_2_^−^ and NO_3_^−^ also decreased at the same time, signaling that AOB and NOB microbial activity was reduced [87]. Dzionek et al. [38] proved that diclofenac caused only minor temporary alterations in NH_4_^+^ oxidation. There may be a noticeable effect at the enzymatic and energetic levels resulting from partial inhibition of the ammonium monooxygenase and disruption of oxidative phosphorylation. These results are consistent with those reported by Ciftcioglu et al. [118]. A mixture of six NSAIDs (including bicyclic compounds such as naproxen, diclofenac, ketoprofen, and mefenamic acid) at a total concentration of 100 µg/L caused 33% and 35% inhibition of the growth of AOB and NOB bacterial consortia, respectively. The effect of bicyclic NSAIDs on nitrification is concentration-dependent. High concentrations cause reorganization of the entire microbial consortium and affect nitrification pathways [110,116,117,124,125]. Kruglova et al. [117] found that, despite the presence of diclofenac in MBR and SBR systems, nitrification did not decrease significantly, and nitrogen removal efficiency remained high at approximately 98%. At the same time, the quantity of typical AOB and NOB was limited to around 0.5%. This means that other microbial species took over their primary role in nitrogen oxidation. No significant effect on the process of nitrification under SBR conditions in the presence of a mixture of paracetamol, ibuprofen, naproxen, and diclofenac was noted by Marchlewicz et al. [114]. Gallardo-Altamirano et al. [71] also reported a high ammonium nitrogen oxidation efficiency of nearly 97%. However, shifts in the structure of the microorganism consortium were noted. Diclofenac’s biotransformation is directly mediated by nitrification and denitrification-related microorganisms, as demonstrated by Osorio et al. [79]. The presence of the drug is related to the formation of reactive nitrogen species and nitrated derivatives, indicating a modification of nitrogen cycle pathways. The synthesis of nitrosod-diclofenac and nitro-diclofenac derivatives has been shown to be strongly correlated with the presence of bicyclic NSAID medicines. Dokianakis et al. [122] showed that, depending on the type of substance, the second stage of nitrification is particularly susceptible to the impacts of specific pharmaceutical substances. Triclosan inhibited NOB activity, although diclofenac had no significant effect on NO2- oxidation. There was evidence of NOB community adaptation and nitrification activity regeneration. This suggests that the biological system could still be prepared to adjust and restore function. In addition to a reduction in cell viability, the presence of pharmaceuticals resulted in oxidative stress (increased SOD activity, decreased SDH) while improving EPS formation [87,110]. Additionally, bicyclic NSAIDs altered the structure of microbial population [87,110,116,117,118,123,126,127]. Jiang et al. [110], after exposing the bacterial consortium to 5 µg/L of diclofenac and naproxen, observed disturbances in cellular energy, as evidenced by decreases in succinate dehydrogenase (SDH) activity and increases in superoxide dismutase (SOD) activity and EPS production. These findings suggest that oxidative stress occurred and protective processes were activated. Furthermore, the drugs resulted in a loss of cell membrane integrity. Similarly, Zhao et al. [87] found that at a concentration of 2 mg/L diclofenac, there was a 1.7 times increase in EPS content and a rise in reactive oxygen species (ROS) generation. Phosphorus metabolism-related enzyme activity also declined, with exopolyphosphatase (PPX) and polyphosphate kinase (PPK) declining by approximately 20 percent. There was an increase in LDH (lactate dehydrogenase) release, which may indicate that the cell membrane’s structural integrity has been disturbed. At lower, environmentally relevant concentrations (1 µg/L), no changes in phosphate removal or a decrease in chemical oxygen demand (COD) removal effectiveness were observed [87]. Also, at lower concentrations, no decrease in nitrogen removal efficiency was observed [111,114,117,118], including changes in OUR [118], biomass [77,111,118], sludge structure [38,111,128], or the basic physicochemical indicators of the activated sludge [111,118]. Exposure of activated sludge to bicyclic drugs did not significantly affect the removal of PO_4_^−^ or COD, but it did reveal differences in the metabolic potential of the microbial community [87,110,117]. Diclofenac in a dose of 1 μg/L did not significantly affect these parameters [117]. In Ciftcioglu et al.’s [118] study, no changes in COD removal were observed. The presence of bicyclic NSAIDs in the active sludge leads to changes at the enzymatic level [116,119]. It was shown that exposure to diclofenac induced a remodeling of the lipid composition of cell membranes. They noted an increase in the proportion of saturated and cyclopropane fatty acids, which indirectly means a modification of the activity of enzymes responsible for lipid synthesis and remodeling [116]. The analysis described by Demaria et al. [119] showed an increase in the number of copies of genes encoding monooxygenases, including ammonium monooxygenase and methane monooxygenase.

### 5.5. Effect of Bicyclic NSAIDs on the Structure of the Activated Sludge Microbiome

The presence of NSAIDs can act as a selection factor, leading to the elimination of susceptible populations and the promotion of microorganisms capable of adapting. Exposure to bicyclic NSAIDs at 0.5 mg/L consistently reduces biodiversity and selectively enriches resistant taxa, as confirmed in both classical active sludge and constructed wetland systems [126,129].

Confirmation of the actual taxonomic rearrangement is provided by the studies of Zhao et al. [87], in which the analysis of 16S rRNA showed a significant reorganization of the activated sludge microbiome under the influence of bicyclic NSAIDs. The share of *Proteobacteria* decreased from 76.92% to 51.31%, while the share of *Bacteroidetes* increased from 3.92% to 34.23%, with the simultaneous disappearance of Firmicutes. Prior to exposure, *Betaproteobacteria* (48.3%), *Epsilonproteobacteria* (20.8%), and *Clostridia* (18.1%) dominated; however, with the introduction of drugs, a decrease in Betaproteobacteria and an increase in the proportions of *Sphingobacteria* and *Alphaproteobacteria* were observed. After a period of adaptation, the structure of the microbiome stabilized, indicating the ability of the community to adapt to new environmental conditions. The decrease in the share of *Betaproteobacteria*, bacteria responsible for the processes of removing organic compounds and nitrogen in activated sludge, indicates their high sensitivity to bicyclic NSAIDs. In turn, the increase in the share of *Bacteroidetes*, including *Sphingobacteria*, indicates the selection of organisms capable of decomposing complex organic compounds, and that group is more adapted to environmental stress. The increase in *Alphaproteobacteria* may indicate the predominance of microorganisms with high tolerance to toxic compounds. The decrease in *Firmicutes* numbers suggests that this group was susceptible to bicyclic NSAIDs. Given the involvement of *Firmicutes* in the degradation of cyclic compounds in sludge, the changes occurring under the influence of bicyclic NSAIDs may lead to a reduction in the degradative activity of activated sludge [87]. The research of Chonova et al. [74] indicates that the nature of the wastewater inflow significantly affects the structure of the activated sludge microbiome. It was shown that species diversity was higher in urban wastewater than in hospital wastewater. The analysis of OTUs (Operational Taxonomic Units) and the Shannon index showed higher values in urban wastewater consortia than in hospital consortia, suggesting that a higher pharmaceutical burden may lead to a reduction in microbial diversity. On the other hand, Silva et al. [130] confirmed the dependence of the effect on diclofenac concentration, which, at concentrations above 10 mg/L, inhibited acetogenic bacteria and acetoclastic methanogens, indicating selective sensitivity of specific functional groups and potential shifts in the structure of the microbial community. Similar relationships were also observed by Yang et al. [131], who showed that diclofenac was only partially degraded during anaerobic digestion of the sludge, while inhibiting hydrolysis, acidogenesis, and acetogenesis, and reducing the number of bacteria responsible for hydrogen production, indicating the selective sensitivity of bacteria performing key fermentation functions. Exposure to bicyclic NSAIDs leads to a reorganization of the microbiome towards the enrichment of microorganisms capable of detoxification and commetabolic degradation of drugs [127]. However, Nguyen et al. [77] demonstrated that the presence of diclofenac at concentrations of 50–5000 µg/L did not cause a noticeable disturbance in the structure of activated sludge microorganism consortia; however, changes were observed in the proportions of some bacterial groups. There were no shifts in phylogenetic structure, and the Shannon index did not change, indicating that biodiversity in activated sludge remained unchanged with and without this drug. The numbers of *Actinobacteria*, *Gammaproteobacteria*, and *Deltaproteobacteria* in activated sludge increased, while the proportions of *Alphaproteobacteria* and *Cytophagi* decreased as the diclofenac concentration increased. Taxa such as *Nitratireductor*, *Pseudoxanthomonas*, and *Asticcacaulis* were noticed at the highest concentration. As Nguyen et al. [77] point out, *Nitratireductor* are detected in polluted water because they can detoxify organic compounds. *Pseudoxanthomonas*, on the other hand, may have more specialized metabolic processes for dealing with xenobiotics than other bacteria. Microbiome reorganization is therefore common following bicyclic NSAIDs exposure, with constant biomass levels and constant biodiversity rates [128]. Sometimes, the intense selection of the microbiome caused by drug presence can also promote pathogen growth in the aquatic environment [123]. A comparison of the results of Jiang et al. [110] and Kruglova et al. [117] indicates that although both studies confirm the significant impact of bicyclic NSAIDs on the remodeling of the activated sludge microbiome, they differ in their interpretations of the roles of individual bacterial groups and the mechanism of diclofenac removal. The authors of both works indicated that sludges were dominated by *Proteobacteria*, *Actinobacteria*, and *Bacteroidetes*, and the presence of drugs led to taxonomic changes. Moreover, Jiang et al. [110] observed that due to the presence of diclofenac and mixtures of diclofenac with ibuprofen, and diclofenac with ibuprofen and naproxen, the biodiversity index increased with the presence of pharmaceuticals. At the taxonomic level, a noticeable reorganization of the microbiome was observed, with a decrease in the occurrence *Proteobacteria* by approximately 10%. *Actinobacteria*, on the other hand, increased from 29.38% to 45.56%, and *Bacteroidetes* from 6.65% to 9.71%. In addition, Jiang et al. [110] reported that the occurrence of *Actinobacteria* was correlated with increased EPS. In the same work, pharmaceuticals reduced the proportions of genera such as *Micropruina* and *Nakamurella*, and increased the proportions of *Zoogloea*, *Desulfovibrio*, and *Desulfobulbus*. Species whose presence in conditions of exposure to pharmaceuticals increased are involved in the processes of storage of reserve compounds, binding to xenobiotics, and reduction of sulfates, which explains their presence and tolerance to the action of pharmaceuticals in the sludge. The same conclusion was indicated by Kruglova et al. [117], in which an increase in the number of taxa such as *Terracoccus*, *Terrabacter*, *Luteococcus*, *Dechloromonas*, or *Microbacterium* has been identified in reactors with drugs, which may indicate their involvement in the degradation of aromatic compounds or the use of cell decay products. *Proteobacteria*, *Actinobacteria*, and *Bacteroidetes* are key functional groups in activated sludge, and their presence correlates with the degradation of organic matter and various xenobiotics. However, their increased share results from selection and stress effects on the microbiome [125]. The decrease in *Proteobacteria* in the presence of drugs is evidence that the bacteria involved in the nitrogen removal process are sensitive to sudden pharmaceutical stress but can adapt to these conditions [117,125].

To better summarize the diversity of biological wastewater treatment systems and the reported bicyclic NSAID-induced effects, comparative Table 3 and Table 4 of structural and functional changes are presented below.

## 6. Conclusions and Future Perspectives

A systematic, critical literature analysis conducted in accordance with the PRISMA guidelines led to the selection of 31 experimental publications on the effects of bicyclic NSAIDs on biological wastewater treatment systems. The high number of excluded papers and strict inclusion criteria confirm that the available data are limited, and the literature identified is of high quality and strongly focused on the relationship between bicyclic NSAIDs and the functioning of biological treatment systems.

The widespread use of bicyclic NSAIDs and their poor transformation in organisms and the environment result in their appearance in wastewater treatment plants, where they can affect the microbiome of biological treatment systems. However, the extent of these effects varies considerably between studies due to differences in experimental design, reactor configuration, operating conditions, exposure time, and drug concentrations.

The studies reviewed suggest that bicyclic NSAIDs can modify the activity of activated sludge systems, although the observed responses appear to be strongly dependent on the technological configuration and operating parameters. These factors likely play a significant role in microbial adaptation and treatment efficiency. Although the drugs themselves may contribute to changes in microbial activity, their effects are often difficult to separate from those of process conditions. Bicyclic NSAIDs at environmental concentrations do not significantly interfere with aerobic processes, although they may temporarily prolong the intensive oxygen consumption phase, indicating an adaptive metabolic cost incurred by microorganisms in the presence of xenobiotics. The presence of bicyclic NSAIDs, especially at elevated concentrations or prolonged exposure, induces oxidative stress in the cells of activated sludge microorganisms, as evidenced by increased antioxidant enzyme activity and decreased respiratory enzyme activity. In addition, lower concentrations of bicyclic NSAIDs generally do not significantly interfere with nitrogen metabolism, whereas higher concentrations may partially inhibit nitrification and lead to nitrite accumulation. Biological phosphorus removal processes, like other purification processes in wastewater treatment plants, show high resistance to low concentrations of bicyclic NSAIDs, whereas at higher loads of pharmaceuticals, their weakening is observed, associated with increased EPS production and reorganization of the activated sludge microbiome. Importantly, reductions in biomass or activity of sewage sludge were generally reported as temporary rather than permanent effects.

The literature indicates that the presence of bicyclic NSAIDs in the sewage treatment plant inflow leads to qualitative changes in the structure of bacterial communities, including decreases in the shares of some groups (*Firmicutes*, *Proteobacteria*) and increases in others (*Actinobacteria*, *Bacteroidetes*, *Alpha*-, and *Gammaproteobacteria*). These observations suggest the possibility of selective environmental pressure and adaptation of microbial communities. However, due to significant heterogeneity in experimental conditions and analytical methods, it was not possible to identify consistent microbial consortia that were particularly sensitive to bicyclic NSAID-induced structural changes.

Bicyclic NSAIDs do not cause significant destabilization of the macrostructure of activated sludge flocs but modify their microstructure through the increase in EPS production, changes in electrostatic interactions, and the sorption surface, which promotes the adsorption of pharmaceuticals and the reorganization of metabolic niches under xenobiotic pressure.

Most of the cited studies indicate that initial disturbances in biological activity, microbiome structure, or cellular metabolism are compensated over time by the adaptation of microbial populations.

Analysis of the transformation pathways of diclofenac, naproxen, and ketoprofen indicates that hydroxylation, demethylation, oxidation, reduction, and aromatic ring cleavage are the dominant mechanisms of bicyclic NSAID biotransformation. Many of these reactions are catalyzed by non-specific oxidative enzymes, including ammonium monooxygenase and cytochrome-P-450-related enzymes, highlighting the important role of nitrifying and heterotrophic microorganisms in drug degradation. However, incomplete mineralization and the accumulation of transformation products remain significant environmental concerns, especially because some metabolites may retain biological activity or exhibit increased toxicity. Future research should focus on identifying key microbial taxa and enzymatic pathways responsible for efficient bicyclic NSAID biodegradation under real-world wastewater treatment conditions. Furthermore, greater attention should be paid to the ecological significance and toxicity of transformation products generated during biological treatment. Integrating metagenomics, transcriptomics, and metabolomics with process engineering approaches could significantly advance understanding of microbial adaptation mechanisms and drug biotransformation pathways. Furthermore, future research should focus on long-term exposure scenarios, the cumulative impact of pharmaceutical mixtures, and the role of operational parameters such as sludge retention time, dissolved oxygen concentration, and carbon source availability in shaping treatment efficiency. The development of more resilient and functionally diverse biological systems could contribute to the more effective elimination of drug-resistant agents and improved environmental safety in wastewater.

## 7. Limitations

Despite the comprehensive approach to this topic and the scope of systematic and critical review of literature, it is impossible not to emphasize the limitations that affect the weight, potential, and ability to synthesize conclusions. In this context, one of the main limitations of this work is the limited number of experimental studies that meet the adopted strict inclusion criteria for the analysis. The available number of analyzed research results was relatively small, significantly reducing statistical robustness and limiting the ability to draw quantitative or meta-analytical conclusions about the impact of bicyclic NSAIDs on biological wastewater treatment systems. Although the present review focuses on bicyclic NSAIDs, the literature is dominated by studies concerning diclofenac, which is recognized as a priority pharmaceutical due to its high environmental prevalence and toxicity. This results in a disproportionate amount of available data for this compound, whereas other bicyclic NSAIDs are comparatively less studied owing to their lower environmental relevance. Another limitation was the low repeatability and high heterogeneity of the methodologies used in the analyzed experiments. These differences resulted from both the tests used in the reactor configuration (SBR, MBR, continuous flow systems), the operational parameters of the tests (sludge retention time, hydraulic retention time, redox conditions), the wastewater matrix (synthetic wastewater or actual municipal or hospital wastewater), as well as different exposure scenarios (acute or chronic, single compounds or mixtures). As a result, the compilation and comparison of individual study results were difficult, and the effects observed cannot be unambiguously linked to bicyclic NSAID action alone. Another limitation was the lack of a full reflection of the complexity and buffer capacity of full-scale wastewater treatment plants. Many studies are based on laboratory or pilot systems that do not reflect real-world conditions. As the last factor pointing to a barrier to the potential of this work, we can point to the inconsistency and partial contradiction in the research results presented by the authors. This is particularly important in the analysis of the effects of bicyclic NSAIDs on nitrification inhibition, enzymatic activity, or changes in microbial community structure, where some studies report insignificant or no effects, while others report significant effects. Such results suggest a strong dependence on the local adaptation history of microorganisms, operational conditions, and analysis endpoints. To sum up this aspect of this review, the analyzed area of knowledge remains fragmented, which justifies the need for coordinated, long-term, and full-scale research.

## Figures and Tables

**Table 1:** Chemical formulas, classification of selected bicyclic NSAIDs, and their concentration in wastewater [6,26,27,28,29,30,31,32,33,34,35,36].

Bicyclic NSAIDs Class	Empirical Formula	Structural Pattern	Name of the Active Substance	Concentration in Wastewater
Acetic acid derivatives	C_15_H_12_BrNO_3_	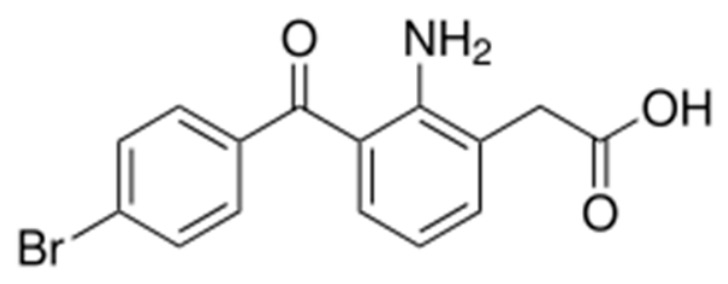	Bromfenac	No data
	C_14_H_11_C_l2_NO_2_	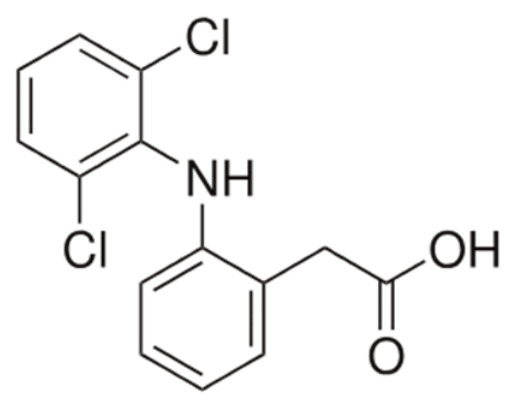	Diclofenak	0.12–68.00 µg/L
	C_14_H_10_C_l2_O_3_	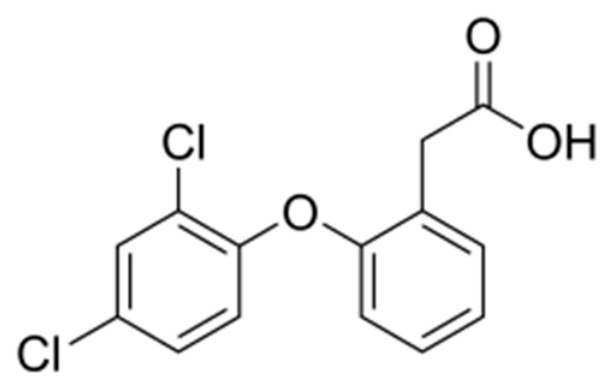	Fenclofenac	No data
	C_15_H_16_O_2_	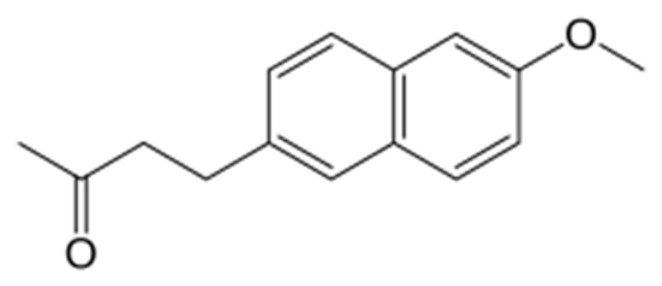	Nabumeton	No data
Anthranilic acid derivatives	C_14_H_10_F_3_NO_2_	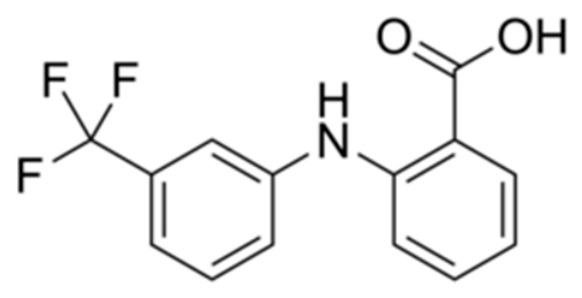	Flufenamic acid	No data
	C_15_H_15_NO_2_	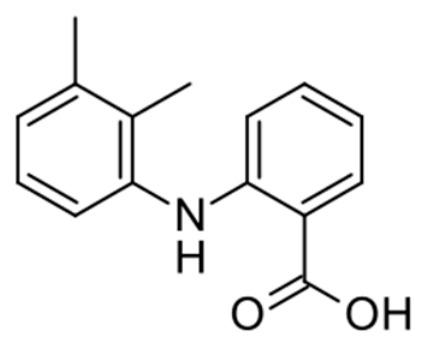	Mefenamic acid	0.005–9.10 µg/L
Phenylacetic acid derivatives	C_16_H_13_C_l2_NO_4_	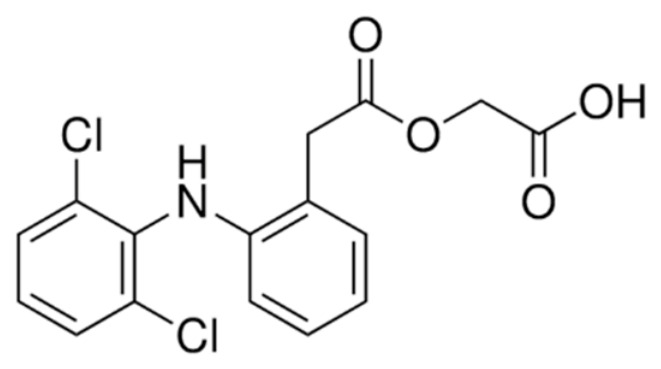	Aceclofenac	0.03 µg/L
	C_15_H_14_O_3_	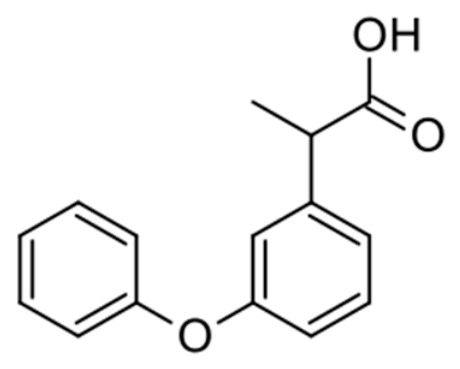	Fenoprofen	0.00–0.76 µg/L
	C_15_H_13_FO_2_	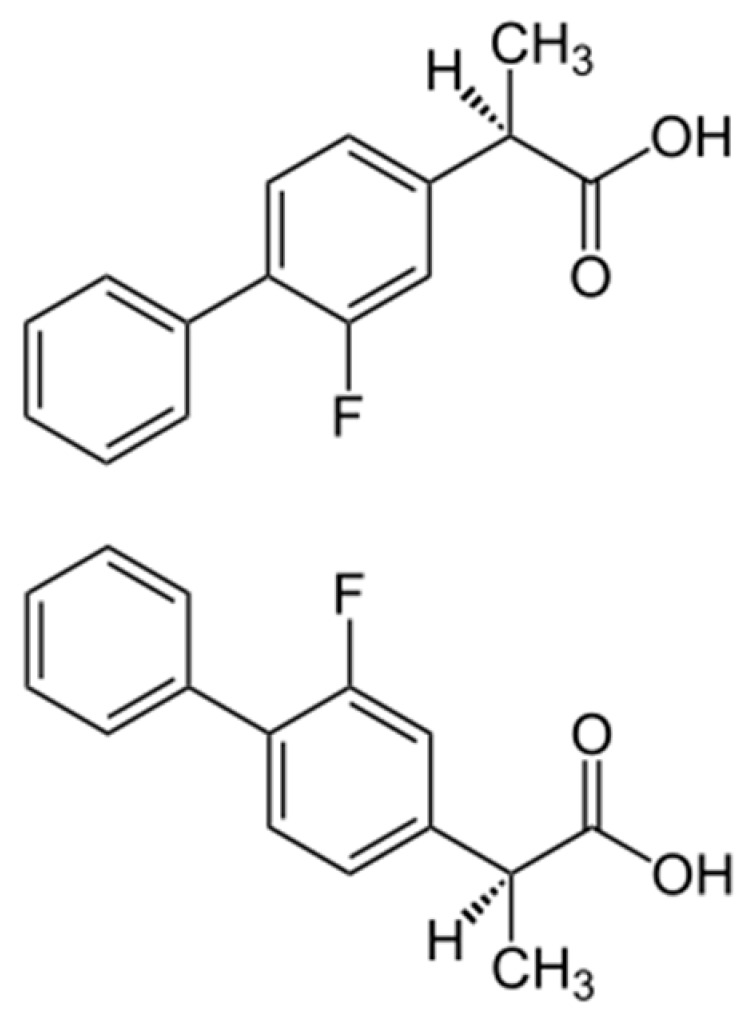	Flurbiprofen	0.01–247.91 µg/L
	C_16_H_14_O_3_	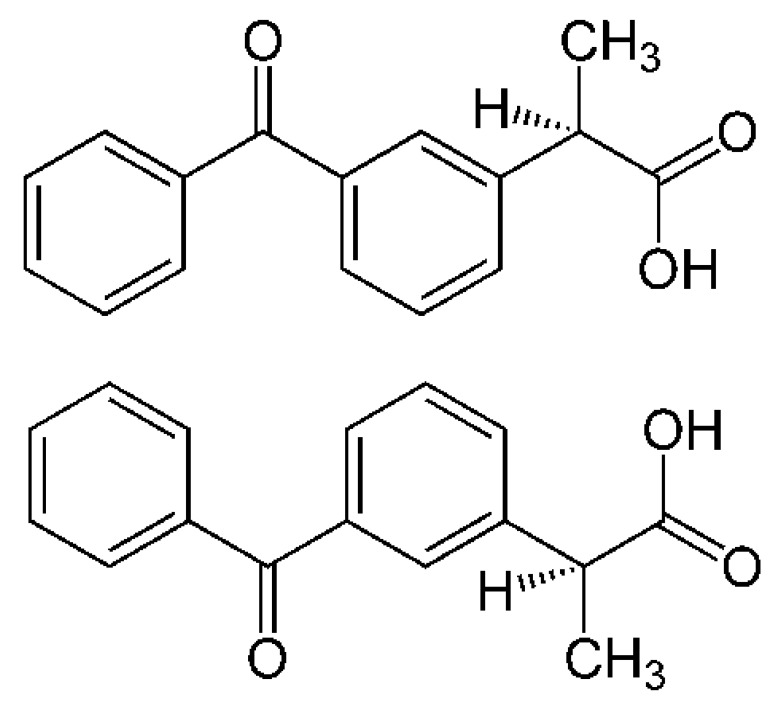	Ketoprofen	0.05–41.00 µg/L
	C_14_H_14_O_3_	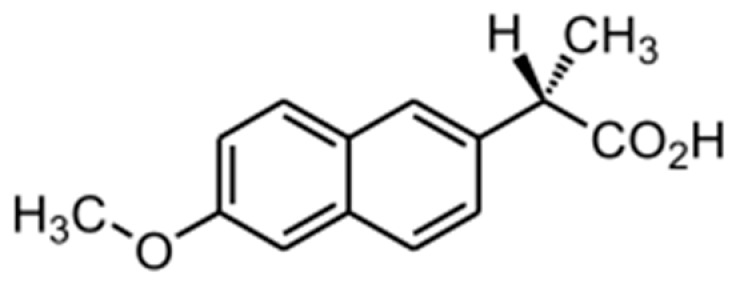	Naproxen	0.08–217.00 µg/L
Sulfonanilides	C_13_H_12_N_2_O_5_	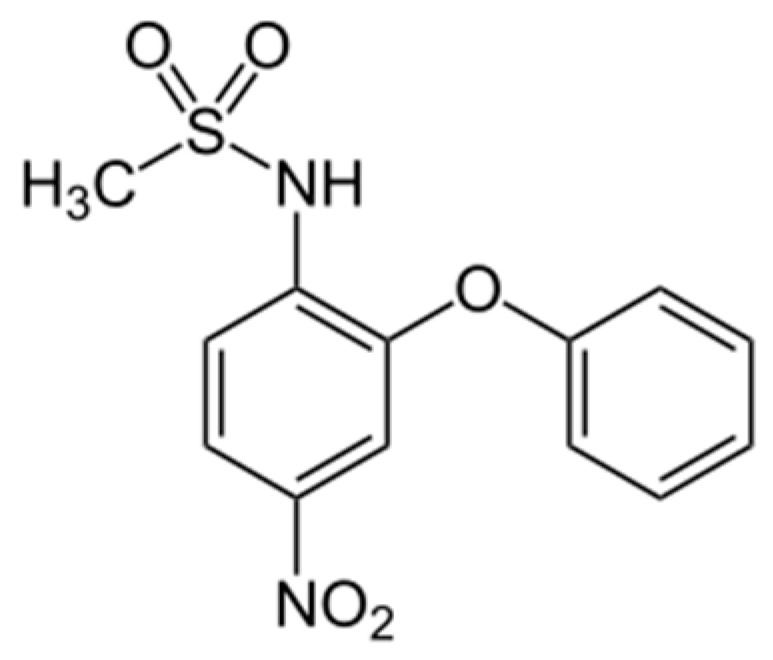	Nimesulid	0.07–1.07 µg/L

**Table 2:** Comparative overview of bicyclic NSAID occurrence in aquatic environments and wastewater treatment plants, including analytical methods, sampling matrices, and treatment system characteristics.

Country/Region	Matrix/Sampling Site	Bicyclic NSAIDs Detected	Concentration Range	Identification/Analytical Method	Sampling Period/Conditions	Type of WWTP/Environmental Context	References
The Czech Republic	Surface rivers (Elbe basin)	Naproxen, diclofenac	Naproxen: 58–160 ng/L; diclofenac: 200–260 ng/L	LC-MS/MS	Seasonal river monitoring	River ecosystems influenced by municipal discharges	[64]
Italy	Surface rivers	Nimesulide, naproxen, ketoprofen	<3–30 ng/L	LC-MS/MS	Spring–summer and autumn–winter 2012	Surface waters characterized by a different anthropic impact	[65]
Poland	The middle stream flow region of the Warta River	Diclofenac, ketoprofen, naproxen, fenoprofen	1.4–2200 ng/L depending on compound and site	LC-MS/MS	The winter, spring, summer periods	Waters impacted by municipal and industrial WWTP effluents	[66]
South Africa	Wastewater and river water	Naproxen, ibuprofen, diclofenac	ng/L–μg/L range	SPE-LC-MS/MS	From January to May in 2016	Municipal wastewater discharges	[56]
Sweden	WWTP influent and effluent	Naproxen, diclofenac	Naproxen: 121–1674 ng/L; diclofenac: 149–196 ng/L	LC-MS/MS	Routine WWTP sampling	Municipal activated sludge WWTP	[68]
Venezuela	Natural waters	Diclofenac, ketoprofen, ibuprofen	Diclofenac: 365–990 ng/L; ketoprofen: 0.18–0.74 μg/L	HPLC-based analysis	Not specified	Municipal wastewater treatment systems	[49]
China (Guangzhou)	WWTP effluent	Diclofenac, naproxen	Diclofenac: 131 ng/L naproxen: 324 ng/L	HPLC/LC-MS	Aerobic activated sludge conditions	Municipal WWTPs	[54]
Turkey	Hospital wastewater	Ketoprofen, naproxen, ibuprofen	Ketoprofen: 9193 ng/L; naproxen: 7186 ng/L; ibuprofen: 1418 ng/L	LC-MS/MS	Seasonal (summer/winter comparison)	Hospital wastewater systems	[71]
Spain	Influent and effluent of pilot-scale WWTP	Diclofenac, naproxen, ketoprofen	166–1748 mg/(1000 inh×d)	LC-MS/MS	Two operational phases	Pilot-scale A2O biological WWTP	[72]
Europe	Conventional WWTPs	Multiple pharmaceuticals, including bicyclic NSAIDs	ng/L–μg/L	LC-MS/MS	Long-term monitoring	Conventional activated sludge WWTPs	[73]
France	Hospital and urban wastewater	Diclofenac, ibuprofen	Not specified	LC-MS/MS	Comparative real-scale study	Separate hospital and municipal WWTPs	[74]

**Table 3:** Comparison of biological wastewater treatment systems used for bicyclic NSAID removal.

System	Main Characteristics	Dominant Biomass Type	Main Removal Mechanisms	Advantages	Limitations	Reported Effect on Bicyclic NSAIDs Removal	References
Conventional activated sludge (CAS)	Suspended biomass in aerated reactors	Floc-forming bacteria	Biotransformation, sorption	Low operational cost, widely used	Limited removal of persistent bicyclic NSAIDs (e.g., diclofenac)	Moderate removal efficiency; dependent on SRT and microbial adaptation	[14,73,85,88,89,90,94,96]
Sequencing batch reactor (SBR)	Cyclic operation with fill–react–settle phases	Activated sludge	Biotransformation, nitrification/denitrification	Flexible operation, stable nitrogen removal	Sensitive to shock loads and pharmaceutical mixtures	Stable process performance despite partial inhibition of bicyclic NSAIDs degradation	[110,113,114,117,120]
Membrane bioreactor (MBR)	Combination of activated sludge and membrane filtration	Suspended biomass with membrane retention	Enhanced biodegradation and biomass retention	High SRT, efficient micropollutant removal	High energy demand and membrane fouling	Improved diclofenac transformation and metabolite removal	[101,111,116,117]
Biofilm systems	Biomass attached to surfaces/carriers	Biofilm-forming microorganisms	Sorption and cometabolic degradation	Higher resistance to toxic compounds	Diffusion limitations inside biofilm	Increased tolerance to pharmaceutical stress and bicyclic NSAIDs improved degradation	[14,85,89,91,92,93]
Granular sludge systems	Dense microbial granules with stratified structure	Aerobic/anoxic granules	Simultaneous nitrification–denitrification and biodegradation	Compact structure, good settling properties	Long start-up period	Potentially improved removal of bicyclic NSAIDs due to microbial stratification	[85,89,107]

**Table 4:** Functional and structural changes in biological wastewater treatment systems exposed to bicyclic NSAIDs.

Type of Change	Observed Effect	Main Affected Microorganisms/Processes	Bicyclic NSAIDs Involved	References
Functional change	Nitrification inhibition	AOB and NOB activity reduction	Diclofenac, ketoprofen, naproxen	[87,118]
	Increased oxidative stress	Activated sludge bacteria	Diclofenac, naproxen	[87,110]
	Increased EPS production	Protective microbial response	Diclofenac	[87,110]
	Reduced methanogenesis	Methanogenic archaea	Diclofenac	[112]
	Altered enzymatic activity	Nitrifiers and heterotrophs	Bicyclic NSAIDs mixtures	[87,98,110]
Structural change	Decrease in *Proteobacteria* abundance	Nitrogen-removing bacteria	Diclofenac	[87,110]
	Increase in *Bacteroidetes* and *Actinobacteria*	Xenobiotic-degrading taxa	Diclofenac, bicyclic NSAIDs mixtures	[87,110,117]
	Reduction in biodiversity	Sensitive microbial populations	Bicyclic NSAIDs	[126,129]
	Selection of resistant taxa	*Pseudoxanthomonas*, *Nitratireductor*	Diclofenac	[77]
	Microbiome reorganization	Activated sludge community	Bicyclic NSAIDs mixtures	[87,117,127]

## Data Availability

No new data were created or analyzed in this study. Data sharing is not applicable to this article.
